# Multidirectional Cylindrical Piezoelectric Force Sensor: Design and Experimental Validation

**DOI:** 10.3390/s20174840

**Published:** 2020-08-27

**Authors:** Ye Rim Lee, Justin Neubauer, Kwang Jin Kim, Youngsu Cha

**Affiliations:** 1 Department of Materials Science and Engineering, Korea Advanced Institute of Science and Technology, Daejeon 34141, Korea; erynlee@kaist.ac.kr; 2 Center for Intelligent & Interactive Robotics, Korea Institute of Science and Technology, Seoul 02792, Korea; 3 Department of Mechanical Engineering, University of Nevada, Las Vegas, NV 89154, USA; neubau10@unlv.nevada.edu (J.N.); kwang.kim@unlv.edu (K.J.K.)

**Keywords:** cylindrical sensor, force sensor, multidirectional, piezoelectricity

## Abstract

A common design concept of the piezoelectric force sensor, which is to assemble a bump structure from a flat or fine columnar piezoelectric structure or to use a specific type of electrode, is quite limited. In this paper, we propose a new design of cylindrical piezoelectric sensors that can detect multidirectional forces. The proposed sensor consists of four row and four column sensors. The design of the sensor was investigated by the finite element method. The response of the sensor to various force directions was observed, and it was demonstrated that the direction of the force applied to the sensor could be derived from the signals of one row sensor and three column sensors. As a result, this sensor proved to be able to detect forces in the area of 225° about the central axis of the sensor. In addition, a cylindrical sensor was fabricated to verify the proposed sensor and a series of experiments were performed. The simulation and experimental results were compared, and the actual sensor response tended to be similar to the simulation.

## 1. Introduction

Piezoelectric materials have received considerable attention in the field of sensing [1,2,3] and energy harvesting [4,5,6] because of their flexibility, fast response speeds, as well as the properties of generating internal electric potential from mechanical deformation [7,8]. In particular, the electromechanical effect of these materials makes it possible to detect physical stimuli without external power, so many researchers have studied various sensors, such as force and strain sensors, using this mechanism [9,10,11,12]. Among these, force sensors have become more important with the development of advanced robots, such as soft robots, humanoid robots, and medical robots [13,14,15,16,17,18]. This is because force sensors allow the robot to undergo dexterous manipulation [19,20,21] or to distinguish objects [22].

These force sensors have been mainly investigated using various materials, such as capacitive [23,24,25], piezo-resistive [26,27,28], and piezoelectric materials [29,30]. Furthermore, many studies have been conducted to apply specific structures to these materials for more efficient force sensing. For example, many studies have been conducted to improve the performance of the sensor by applying various shapes, such as pyramids [31], domes, pillars [32], and springs [33], to resistive materials. However, the most common design concept of piezoelectric force sensors includes assembling a bump structure from a flat- or micro-pillar-shaped piezoelectric structure or to use electrodes of specific shapes [29,34,35]. In other words, the structure of the piezoelectric force sensor is quite limited.

**Figure 1:**
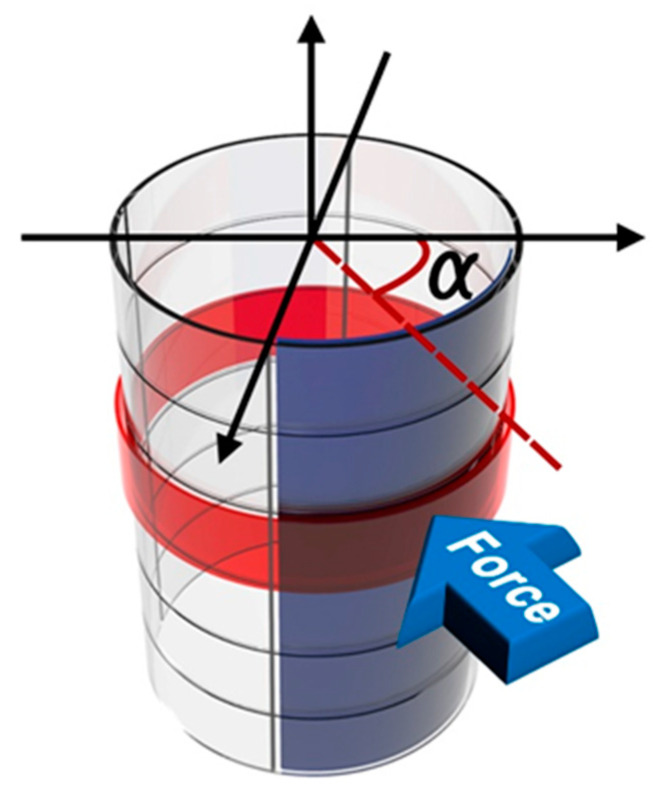
Conceptual diagram of the multidirectional cylindrical piezoelectric force sensor.

Here, we propose a new design of a cylindrical piezoelectric sensor capable of multidirectional force sensing. The sensor has four inner column electrodes and four outer row electrodes. Previous multidirectional force sensors using the piezoelectric effect have a limitation in that the number of electrodes must be greatly increased to increase the resolution, and its wiring becomes more difficult [29,34,35]. However, our sensor can achieve a relatively high resolution with a small number of electrodes. As shown in Figure 1, when a force is applied, the sensor generates column (blue color) and row signals (red color) that correspond to the position where the force is applied. The signals of two adjacent columns to the force applied column are also generated, so that the magnitude of the signal in a total of three columns has a certain tendency according to the angle (a) in which the force is applied about the cylindrical central axis. Thus, we can derive the position where the force is applied. The design of the proposed sensor is explained by finite element method simulation using COMSOL Multiphysics software. In addition, an experiment was performed to verify the proposed model. By comparing the simulation and experimental results, we demonstrated that the proposed new design of the sensor can detect multidirectional forces.

## 2. Simulation

This section describes the simulation for the proposed piezoelectric sensor model. The sensor was modeled using COMSOL 5.4 Multiphysics software with solid mechanics, electrostatics, piezoelectric effect, and electric circuit physics modules.

**Figure 2:**
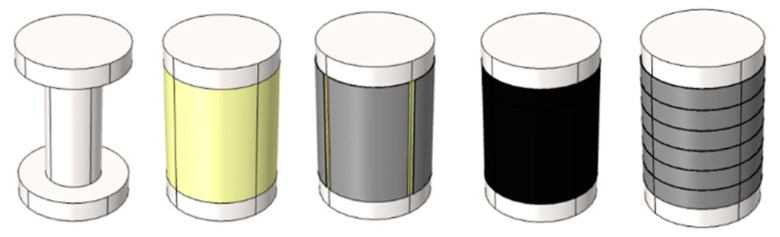
Decomposition of model components showing separate domains, including the 3-D-printed supporter (*white*), PDMS (*yellow*), electrodes (*gray*), and PVDF (*black*) domains.

**Figure 3:**
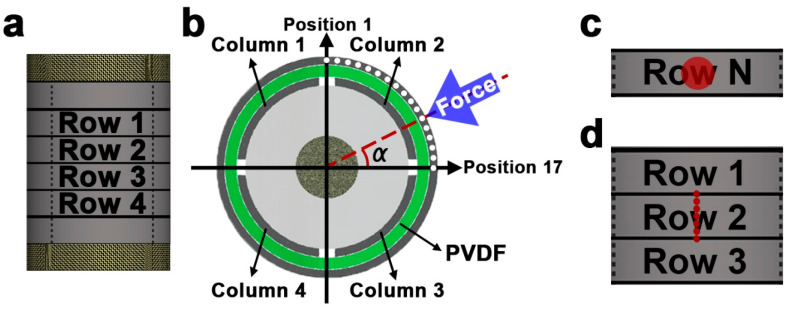
Schematic of the sensor configuration. (**a**) Four row sensors and (**b**) four column sensors with sensor numbers (white circles in column 2 represent the 17 equidistant test points.). The position of the force investigated in the simulation for (**c**) when a force is applied along the lateral midline of each row and (**d**) when a force is applied between the two rows (red circles represent the test points).

Figure 2 shows the deconstructed view of the proposed sensor model domains. The model contains four separate domains with the following material properties: 3-D-printed supporter, poly(dimethylsiloxane) (PDMS), silver electrodes, and polyvinylidene fluoride (PVDF). It should be stated that the model contains four columns and six rows, identical to the physical sensor, with only the middle four rows used for sensing. The top and bottom rows are in contact with the border between PDMS and the 3-D-printed supporter. As a result, the signals from the rows are highly distorted when a force is applied, unlike other rows. Therefore, we only use four rows. For the convenience of discussion, each sensor is named as shown in Figure 3.

### 2.1. Modeling Structure

The solid mechanics module acts as the input for the proposed sensor. The solid mechanics module includes linear elastic materials of all domains, including the 3D-printed supporter, PDMS, electrode, and PVDF domains. The forces are applied to the surface of the outer electrode domain distributed on a circular surface to simulate the cylindrical plunger in experimental work. The base of the sensor was assigned a fixed boundary condition while the remaining boundaries are free. The simulations were performed for multiple positions of forces. First, the forces of 1 N with a speed of approximately 70 mm/min were applied and released along the lateral midline of each row as shown in Figure 3c. Additionally, the position of the applied forces began at the boundary between the column 1 and 2 and terminated at the boundary between the column 2 and 3. A total of 17 equidistant forces were tested from the point where a = 90° to the point where = 0° as shown in Figure 3b, in other words forces were tested at each 5.625° within the column 2. Because the structure of the sensor is symmetrical, no other columns were investigated. Finally, it was investigated whether the position of the force can be detected even when a force is applied between the two rows as shown in Figure 3d. The section from the center of row 1 and row 2 to the center of row 2 and row 3 was divided into six equal parts, and then a force of 1N was applied to the seven positions as shown in Figure 3d. Likewise, due to the symmetry of the sensor structure, no other rows were investigated. Additionally, all forces tested in the model were directed towards the center of the proposed sensor limiting shear forces as shown in Figure 3b.

The electrostatics module begins with a user-defined coordinate system in order to take the PVDF polarity into account. The piezo-strain matrix was applied identically to the piezoelectric film used in the experiment as follows: d_31_ and d_33_ are 23 × 10^−12^ and 33 × 10^−12^ m/V, respectively. The piezoelectric effect couples the electrostatics and solid mechanics modules for the domains containing piezoelectric material. This reflects the charge generated by the PVDF strain.

The electric circuit module treats each electrode terminal as a voltage source. These sources are then connected to a 50 MW load resistor. The total output of the time-dependent model is the measured voltage potential across this resistor.

### 2.2. Simulation Results and Discussion

**Figure 4:**
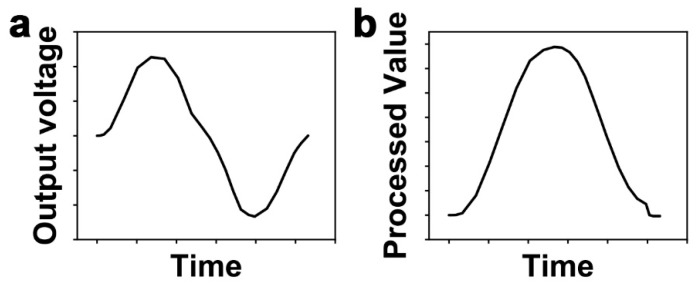
Schematic diagram of an example of a sensor signal when a force is applied and released. (**a**) Output voltage of a single sensor. (**b**) Processed value from the output voltage of a single sensor.

When a force is applied and released to the piezoelectric sensor, the peak value of the output voltage of the sensor is proportional to the speed of the applied force [36,37]. That is, the peak value of the output voltage of the piezoelectric sensor depends not only on the magnitude of the applied force but also on the speed. Therefore, we analyzed the behavior of the sensor by processing the output voltage of the sensor as a value that is independent of the speed of the applied force and proportional to only the magnitude of that. The processed value was calculated by the following equation:

(1) $$\;\;I_{n}=I_{n-1}+V_{n}\times ∆t,$$

where $I_{n}$ is the processed value, $V_{n}$ is the output voltage without an offset, and ∆t is the sensing time interval. That is, the processed value is the integration value of the output voltage of the sensor over time (Figure 4). Here, the output voltage is proportional to the speed of the applied force, so the processed value after the integration is proportional to the displacement, which is proportional to the magnitude of the applied force, not the speed. Then, these processed values were normalized for comparison with the experimental results in Section 3. The processed values are normalized based on the largest magnitude of the responses for all positions, and the row and column signals are normalized individually.

**Figure 5:**
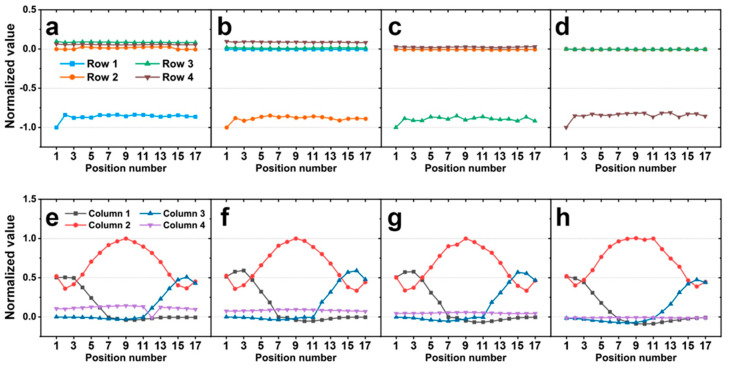
Simulation results of the normalized value from the output voltages of a sensor (**a**,**e**) when 1 N of force was applied to row 1, (**b**,**f**) when 1 N of force was applied to row 2, (**c**,**g**) when 1 N of force was applied to row 3, and (**d**,**h**) when 1 N of force was applied to row 4. The graphs above show the response of the row sensors, and the graphs below show the response of the column sensors.

Figure 5a–d shows the simulation results when the force is applied to row 1, 2, 3, and 4 in order. Likewise, Figure 5e–h shows the responses from column 1–4 when the force is applied to row 1–4. The results show a few apparent trends as shown. First, the row response only shows the signal of the row on which the force is applied, and the responses of other rows are negligible. Additionally, the row response remains nearly constant and is seemingly independent of the force position (Figure 5a–d). This reflects the forces being coincident with the horizontal midline of each row during loading. In other words, the vertical position of the force is not changing within the row, resulting in a constant row response. Thus, the row response can be used to detect the row to which the force is applied. A second noticeable trend is the response of the column 2. Column 2 has a maximum response when loading is in the center of column 2 (Figure 5e–h). When the applied force position is moved away from the center of column 2, the response of the column 2 is decreased. Finally, when the force is applied near the column 1 or 3, the respective column will have an increased response. This indicates that the position of the applied force can be determined to some extent using the three column signals. Herein, we observed the difference in the signals in columns 1 and 3 to make this clearer.

**Figure 6:**
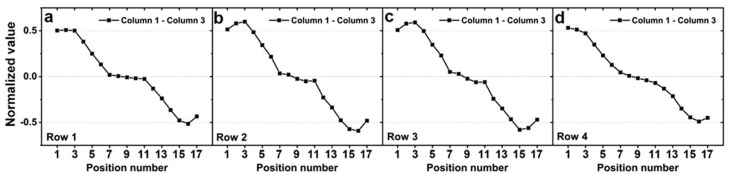
Simulation results of difference between normalized values for column 1 and 3 (**a**) when 1 N of force was applied to row 1, (**b**) when 1 N of force was applied to row 2, (**c**) when 1 N of force was applied to row 3, and (**d**) when 1 N of force was applied to row 4.

As shown in Figure 6, the differences in the signals in columns 1 and 3 tended to decrease, regardless of which row had the force applied. Because the signals in column 2 are symmetrical with respect to the center of the column, the signals in column 2 alone cannot distinguish between positions 1–9 and 9–17. However, since the signal differences in columns 1 and 3 tend to decrease from position 1 to 17, the position of applied force can be distinguished using these signals.

**Figure 7:**
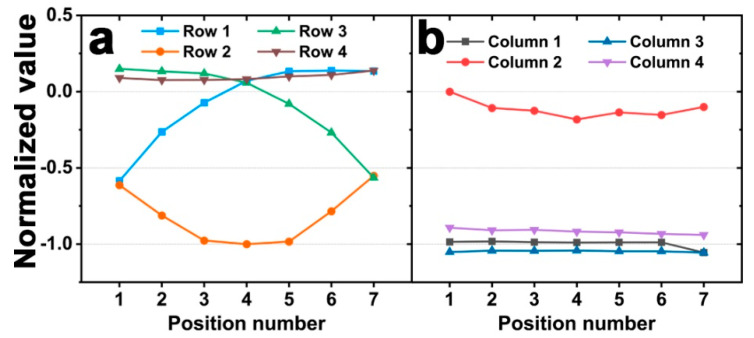
Simulation results of the normalized value from the output voltages of a sensor when 1 N of force was applied between the center of row 1 and 2 to row 2 and 3 in column 2 at (**a**) the row parts and (**b**) column parts.

Finally, we confirmed that this sensor could detect the position of a force applied between two rows rather than the center of a row. In column 2, the section from the center of row 1 and 2 to the center of row 2 and 3 was divided into six equal parts, and 1 N force was applied to the seven positions. First, when the loading is in the center of row 2, row 2 has the maximum response (Figure 7a). If the position of the applied force moves away from the center of row 2, the response of row 2 is decreased. Additionally, when the force is applied near the center of row 1 and 2 or the center of Row 2 and 3, the response of each row increases. As shown in Figure 7b, the column response is similar to the previous results, the column to which the force is applied has the maximum response, and the responses of the other column are negligible. In addition, the column response remains almost constant. These results suggest that our sensor can detect the force applied to any row position as well as the force applied to the center of each row within the same column.

The mesh size was altered to ensure the mesh size during the study was sufficient to provide accurate results. The mesh size of the preceding data was run with a minimum element size of 800 µm consisting of 82,820 domain elements and 35,383 boundary elements. The mesh density was increased as shown in Table 1 to include more elements, and the sensor outputs for those conditions were analyzed. The normalized value results from differing mesh densities are shown in Table 2 with the input to the models being the first force input on row 1. The mesh refinement study shows very little error in integrated values for row 1 and columns 1 through 3. These values are shown because of their importance in analysis of the directional force detection. The difference in the row 1 integrated values between the original mesh size and the extremely refined mesh size was 10%. The difference between the normalized values in this mesh sensitivity analysis show that the proposed mesh size is sufficient, and these differences may also be attributed to other simulation variables, such as solver steps. This ensures the accuracy of the model with respect to the mesh density.

At this time, there are regions in the proposed sensor that cannot distinguish the position of the applied force. They are areas where the signal in column 2 is smaller than the signal in column 1 or column 3. In order to use the difference between the signals in columns 1 and 3, we must first derive that the force is applied to column 2. When the signal in column 2 is greater than the other column signals, we can derive that the force is applied to column 2, but when it is not, we cannot detect which column the force is applied to. Therefore, when the signal in column 2 is smaller than the signal in column 1 or 3, the position of the force cannot be derived. In other words, since the signal of column 2 is smaller than that of the other columns in the areas of position 1 to 3 and position 15 to 17 in row 1 and 4 (Figure 5e,h), the position of the applied force cannot be determined in the region of about 11.25° away from the boundary with the other columns. Similarly, since the signal of column 2 is smaller than that of the other columns in the areas of position 1 to 4 and position 14 to 17 in row 2 and 3 (Figure 5f,g), the position of the applied force cannot be determined in the region of about 16.875° away from the boundary with the other columns. The reason why the signal in column 2 in these areas is smaller than column 1 or 3 could potentially be described by the curvature changes in each respective column based on the PVDF polarity and will potentially be explored more in the future. In the end, since the signal of column 2 is always larger than that of other columns only between position 4 and 14 for all rows, the area where the proposed sensor in column 2 can distinguish the position of force is about 56.25°. Since this sensor has a symmetrical structure, the total sensing range of the sensor is 225 degrees, which is 4 times the sensing range of column 2.

In summary, the position where the force is applied can be derived in the following three steps: (1) Find out in which row the force is applied by the largest row signal; (2) find out which column the force is applied to by the largest column signal; and (3) by subtracting the signals of the two columns adjacent to the column found in step 2, find out the angle ( ) of the force applied about the cylinder axis.

## 3. Experimental Validation

This section provides the validity of the proposed sensor design. First, the method of fabricating the cylindrical sensor according to the proposed sensor design and the experimental setup are introduced. The data obtained on this basis were compared with the simulation data obtained in the previous section.

### 3.1. Fabrication of Cylindrical Piezoelectric Force Sensor

**Figure 8:**
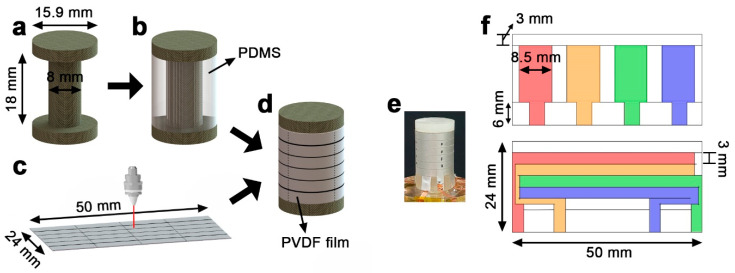
Schematic diagram showing the fabrication process of the cylindrical piezoelectric force sensor. (**a**) 3-D-printed supporter. (**b**) PDMS with a 3-D-printed supporter. (**c)** Electrode patterning of a silver-coated PVDF film. (**d**) Completed sensor with patterned PVDF film. (**e**) Photograph of the fabricated sensor. (**f**) Electrode pattern of the PVDF film (top) inside and (bottom) outside. Different row and column sensors are identified by different colors.

We fabricated the cylindrical piezoelectric sensor using a commercial PVDF film coated with silver electrodes (thickness: 28 m; Measurement Specialties, Inc., Norristown, PA, USA) and PDMS (SYLGARD 184 A/B, Dow Corning Corp., Midland, MI, USA). First, the supporter and mold of PDMS were fabricated using a 3-D printer (ProJet HD3500 Plus, 3D Systems, Inc., Rock Hill, SC, USA) using VisiJet M3 Crystal and VisiJet S300 (3D Systems, Inc., USA) as the build and support materials, respectively. Figure 8a shows the supporter of PDMS. There is a cylinder with a diameter of 15.9 mm located on the top and bottom of a circular pillar with a diameter of 8 mm and a height of 18 mm. After that, the 3-D-printed mold was used to fabricate the PDMS with an outer diameter of 15.9 mm as shown in Figure 8b, so that the cylinder with the 3-D-printed supporter and PDMS had a diameter of 15.9 mm. As shown in Figure 8c, the top and bottom electrodes of the PVDF film with a size of 50 × 24 mm^2^ were patterned in different designs by a laser marking machine (Cat-Fs20 Mini, Marc Co., Ltd., Daegu, Korea). This patterned film is attached to the PDMS portion of the cylinder made earlier (Figure 8d). The completed sensor is shown in Figure 8e. The patterning design of the top and bottom electrodes of the PVDF film is shown in Figure 8f. In the fabricated sensor, the inner electrodes of the PVDF film are composed of electrodes in the form of vertical lines for external wiring, as shown in the upper portion of Figure 8f, and the width of the electrodes used for sensing is 8.5 mm. The outer electrodes of the PVDF film are composed of electrodes in the form of horizontal lines as shown in the bottom of Figure 8f, and the height of the electrodes used for sensing is 3 mm. As a result, the sensor consists of four row sensors and four column sensors. Each row sensor is marked with 32 points at equal intervals. In other words, these points are spaced 11.25° about the central axis of the cylinder. We comment that this distance is twice than the simulation. It is because of the limitation of the enforced point size in the real test. 

### 3.2. Experimental Setup

**Figure 9:**
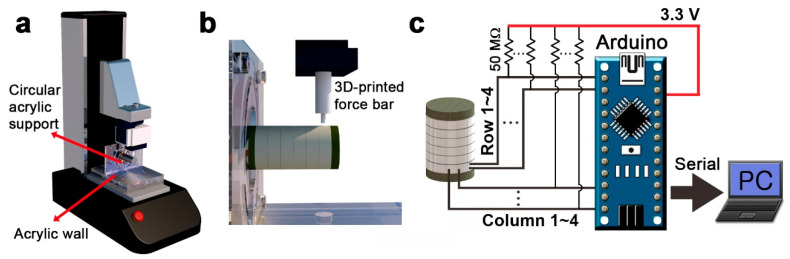
Schematic of the experimental setup. (**a**) Schematic of the tensile testing setup with the sensor using (**b**) a 3-D-printed force bar. (**c**) Schematic of the sensing system in real time.

To evaluate the multidirectional sensing capability of the piezoelectric cylindrical sensor, we applied a constant force to the sensor using a tensile testing machine (MCT-2150, A&D CO., LTD., Tokyo, Japan) after fixing the sensor to a custom-made acrylic supporter consisting of a circular support and an acrylic wall. Figure 9a shows an experimental setup with a sensor mounted on a circular acrylic support. The tensile testing machine is connected to a 3-D-printed force bar with a 1-mm-radius tip as shown in Figure 9b, which applies a force perpendicular to the sensor. The force was applied at a speed of approximately 70 mm/min, and the sensing behavior was investigated for forces of 3, 4, and 5 N. We performed the experiment on only column 2, the same as the simulation.

The output voltages of the sensor were measured by the setup as shown in Figure 9c. Four row and four column sensors are connected to an Arduino Nano microcontroller to read a total of eight analog voltages. Since the sensor has the piezoelectric property, electric power was not applied to the sensor, and only the voltage generated from the sensor was read. Each row and column sensor were connected to a load resistor of 50 MW with an applied offset of 3.3 V to measure the signal in the positive value region. The signal was measured at a sampling rate of 50 Hz and was read into a personal computer via serial communication. In data processing, the offsets of the signal were removed, and the power source noise of 60 Hz was eliminated by a low-pass filter. In addition, as in the simulation, the output voltages of sensor were normalized after processing according to Equation (1).

### 3.3. Model Validation

**Figure 10:**
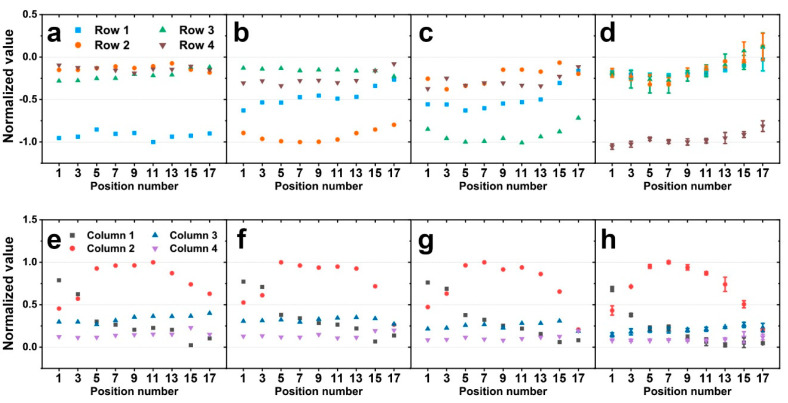
Experimental results of the normalized value from the output voltages of a sensor (**a**,**e**) when 5 N of force was applied to row 1, (**b**,**f**) when 5 N of force was applied to row 2, (**c**,**g**) when 5 N of force was applied to row 3, and (**d**,**h**) when 5 N of force was applied to row 4. The graphs above show the response of the row sensors, and the graphs below show the response of the column sensors. In (**d**,**h**), the error bars are standard deviations.

First, in column 2 of the sensor, the response at each position was observed when 5 N of force was applied to different rows (Figure 10). Here, we performed five trials for each position in row 4 to verify the repeatability of the sensor. As a result, as shown in Figure 10d,h, the sensor response generally has small deviations. Therefore, Figure 10 plots show only one experimental value.

Figure 10a–d shows when forces are applied to row 1, 2, 3, and 4, respectively. As in the simulation, the magnitude of the response in the row in which the force is applied has the largest value, which is almost constant. In the simulation, the rest of the row responses showed negligible values, whereas the experimental responses of row 2–4 showed significant values. This is expected to be caused because the design of the electrode of the actual sensor differs from that of the simulation model.

Next, the experimental responses of the columns were observed (Figure 10e–h). When a force is applied to column 2 of the sensor, the sensor response is concave with respect to the position number. Additionally, the closer the force is to position 1, the greater the signal in column 1, and the closer to 3, the greater the signal in column 3. These tendencies have good correlation with the simulation results. However, in the simulation, the responses in column 2 are symmetrical about the position 9, while the experimental responses show that the axis of the concave curve is shifted in the position 1 direction. The responses in columns 1 and 3 are also symmetric about position 9 in the simulation but not in the experimental results. In addition, in the simulation, the responses of column 2 at positions 1 and 9 are the same as the response values of column 1 and 3, respectively, but the experimental values are different. These differences may have been influenced by the difference in the electrode design between the simulation model and the actual sensor, as mentioned earlier. Furthermore, while the simulation uses a fully cylindrical PVDF domain, the actual sensor is fabricated by attaching a rectangular PVDF film to the PDMS cylinder, so the PVDF domain shape of the actual sensor is different from the simulation. Therefore, this is expected to cause a difference between the experimental and simulation results.

**Figure 11:**
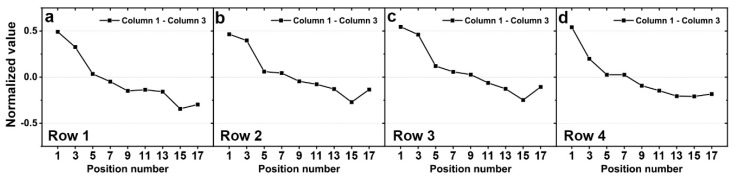
Simulation results of the difference between the normalized values for column 1 and 3 (**a**) when 5 N of force was applied to row 1, (**b**) when 5 N of force was applied to row 2, (**c**) when 5 N of force was applied to row 3, and (**d**) when 5 N of force was applied to row 4.

As in the simulation section, we observed the difference in the experimental response between column 1 and column 3 (Figure 11). Except for position 17, these values tend to decrease in the respective row the force is applied to. The experimental results show differences in the magnitude and symmetry of the simulation and response but show good agreement in terms of tendency. Thus, the proposed sensor can detect the direction of force in positions 3–15 by following the three steps mentioned in Section 2.2.

**Figure 12:**
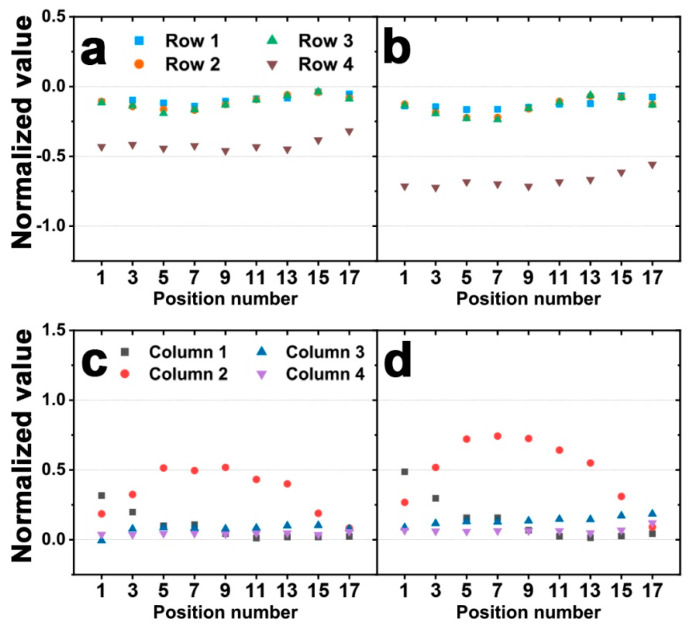
Experimental results of the normalized value from the output voltages of a sensor (**a**,**c**) when 3 N of force was applied to row 4, and (**b**,**d**) when 4 N of force was applied to row 4. The graphs above show the response of the row sensors, and the graphs below show the response of the column sensors.

Finally, we experimented with 3 N and 4 N force applications in row 4 to observe the tendency of signals as the magnitude of the force changed. These signals were normalized to the maximum value of the sensor response when 5 N was applied. Figure 12a,c show the signals when 3 N was applied and Figure 12b,d show the signals when 4 N was applied. As the magnitude of the force decreases, the response magnitude of the sensor also decreases, and the tendency of the sensor response is the same regardless of the magnitude of the force. This result suggests that the proposed sensor can derive not only the direction of the force but also the magnitude of the force.

## 4. Conclusions

In this work, we proposed a new design of a cylindrical piezoelectric sensor that enables multidirectional force sensing. For the demonstration of the sensor design, the responses of the sensor were investigated when a force was applied to each position by the finite element method, and the area where the proposed sensor could detect the direction of the force was observed. The proposed sensor consists of four row and four column sensors that was able to derive the direction of force through one row signal and three column signals. The experimental validity of the cylindrical sensor was verified by comparison with simulation results. The ideal model of the simulation and the fabricated sensor differed in the PVDF domain shape and electrode design, so the experimental results showed that the response magnitude and symmetry differed from the simulation results. However, in terms of tendency, it showed good agreement and proved that the direction of force can be detected in a similar area as the simulation model. In addition, we examined the sensor response according to the magnitude of the force, and the results showed that the proposed sensor has the potential to derive the magnitude of the force. Compared to other curved type sensors, our sensor is not only self-powered but also has a wide angular sensing range and longitudinal resolution (Table 3). This multidirectional force sensor has the potential to be applied in robotics, especially in artificial or robotic finger applications.

## Figures and Tables

**Table 1:** The number of mesh elements.

	Standard Mesh (the Preceding Data)	Fine Mesh	Finer Mesh
Domain elements	82,820	153,217	292,355
Boundary elements	35,383	55,905	103,672

**Table 2:** Simulation results of the normalized value at varied mesh density.

	Standard Mesh (the Preceding Data)	Fine Mesh	Finer Mesh
row 1	1.000	1.020	0.896
column 1	0.504	0.509	0.444
column 2	0.521	0.518	0.436
column 3	0.106	0.117	0.120

**Table 3:** Comparison of sensors on a curved surface.

Characteristics	This Work	[38]	[39]	[40]
Angular sensing range	225°	360°	X	X
Longitudinal resolution	3 mm	X	X	X
Self-powered ability	○	○	○	X
